# Orthohantaviruses infections in humans and rodents in Baoji, China

**DOI:** 10.1371/journal.pntd.0008778

**Published:** 2020-10-19

**Authors:** Hui Tian, Wei-Fang Tie, Hongbing Li, Xiaoqian Hu, Guang-Cheng Xie, Luan-Ying Du, Wen-Ping Guo

**Affiliations:** 1 Baoji Center for Disease Control and Prevention, Baoji, Shaanxi, China; 2 College of Hetao, Bayannur, Inner Mongolia, China; 3 Department of Pathogenic Biology, College of Basic Medicine, Chengde Medical University, Chengde, Hebei, China; NIAID Integrated Research Facility, UNITED STATES

## Abstract

In recent years, hemorrhagic fever with renal syndrome (HFRS) incidence has been becoming a severe public health problem again due to its significant increase in Shaanxi Province, China. Baoji, located in the Guanzhong Plain in the central part of Shaanxi Province, has been severely affected by HFRS since its first emergence in 1955. To better understand the epidemiology of orthohantaviruses infection in humans and the causative agents carried by the rodents, the long-term incidence patterns were analyzed and a molecular epidemiological investigation of orthohantaviruses infection in humans and rodents was performed. During 1984–2019, 13,042 HFRS cases were registered in Baoji, including 275 death cases. Except the first high prevalence of HFRS in 1988–1993, another two epidemic peaks were observed in 1998–2003 and 2012, respectively, although vaccination project was started since 1996. During the same period, HFRS cases in Baoji mainly were recorded in winter suggesting they may be caused by Hantaan orthohantavirus (HTNV), while a small peak of HFRS was also found in summer with unknown reason. Nucleotide identity and phylogenetic analyses demonstrated that a novel clade of HTNV sequences recovered from HFRS cases were closely related to those from rodents, including species close contact with humans, suggesting a direct viral transmission from rodents to humans and the important role in the HTNV transmission the nontraditional rodent hosts may play. Moreover, two distant related Dabieshan orthohantavirus (DBSV) lineages were also identified in *Niviventer niviventer* in this area demonstrating its considerable genetic diversity. Our data indicated that continual spillover of HTNV from rodents to humans, contributing to the high prevalence of HFRS in humans in Baoji.

## Author summary

Hemorrhagic fever with renal syndrome (HFRS) is a serious public health problem and affected Baoji in Shaanxi, China despite onset of free vaccination in populations. Hence, the epidemiology of HFRS was analyzed and a molecular investigation was performed in Baoji. During 1984–2019, a total of 13,042 HFRS cases were recorded and three epidemic peaks were observed. Moreover, 275 deaths were registered, with an average fatality rate of 2.10%. Phylogenetic analysis was indicative of directive transmission of Hantaan orthohantavirus (HTNV) from rodents, including nontraditional rodent hosts to humans because viral sequences obtained from patients in Baoji were very closely related to those from rodents. Our limited data indicated a continual spillover of HTNV from rodents to humans occurred in Baoji, responsible for the periodic high prevalence of HFRS, highlighting the need of continuous surveillance of HTNV in rodents and humans. In addition, two novel Dabieshan orthohantavirus (DBSV) lineages were also identified, suggesting its high genetic diversity.

## Introduction

Genus *Orthohantavirus* (family Hantaviridae), are enveloped, negative-sense, and single-stranded RNA virus. In the past two decades, orthohantaviruses have caused worldwide concern as emerging zoonotic pathogens [1]. Although identified in rodents, insectivores, and bats, only the rodents associated orthohantaviruses are confirmed to be pathogenic to humans and can cause two diseases: hemorrhagic fever with renal syndrome (HFRS) in Asia and Europe, and orthohantavirus pulmonary syndrome in North and South America [1–3]. In contrast, rodent-borne orthohantaviruses establish a persistent infection and cause no obvious harm in their natural hosts [4].

The earliest record of HFRS was in the early 1930s in northeastern China. Since then, China has been affected severely by HFRS because almost 90% cases occur in China [5]. To date, only both rodent-borne Hantaan orthohantavirus (HTNV) and Seoul orthohantavirus (SEOV) are identified to be the causative agents of HFRS in China although more than ten orthohantavirus species within subfamily Mammantavirinae have been detected in rodents, insectivores, and bats [5,6]. In China, HTNV is mainly hosted by the striped field mouse (*Apodemus agrarius*) and SEOV by the Norway rat (*Rattus norvegicus*) and other *Rattus* rats [5]. Molecular and epidemiological evidences showed that HFRS cases occurring in winter mainly were caused by HTNV, while those cases occurring in spring and summer mainly by SEOV [7].

**Fig 1:**
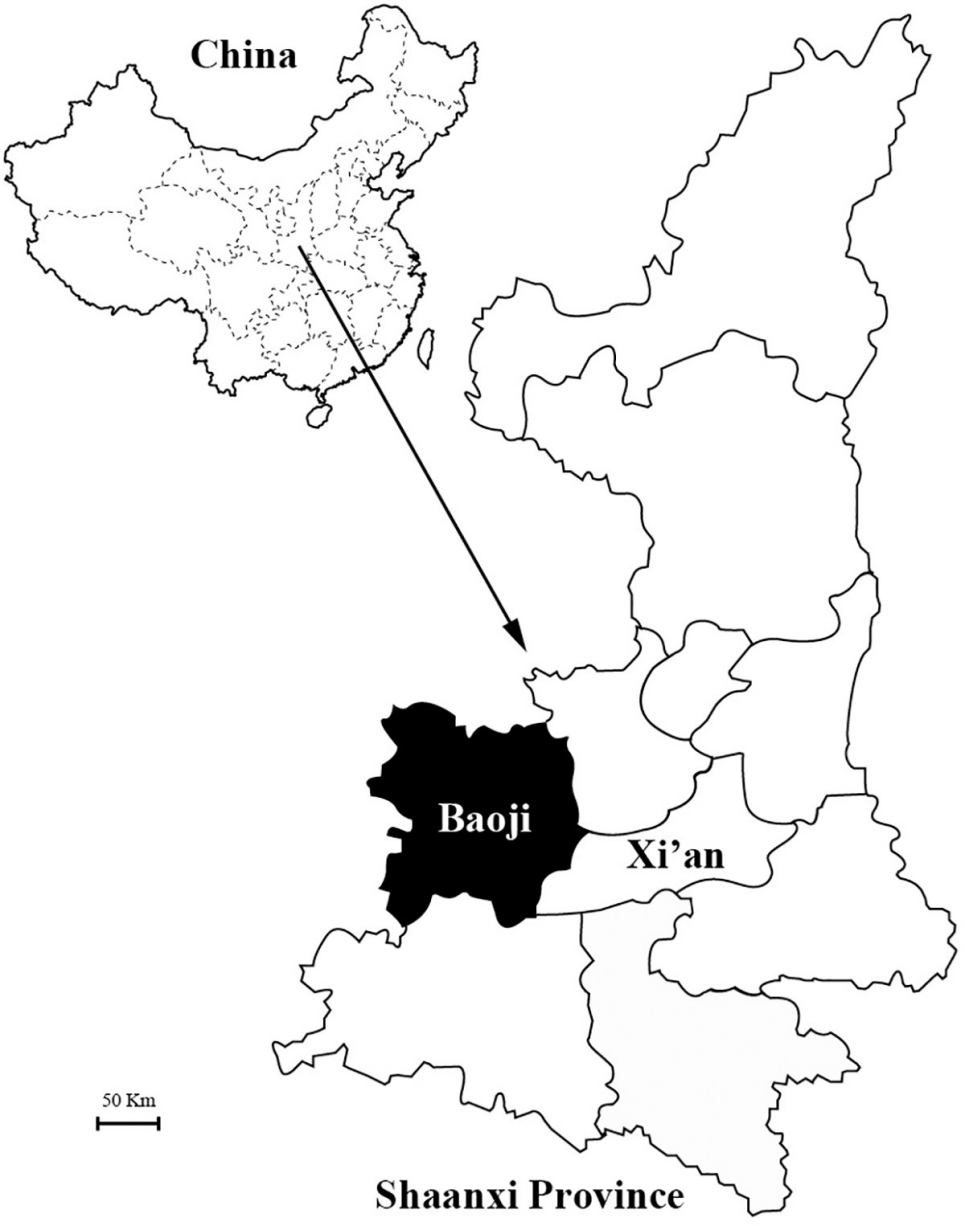
Map of Baoji city in Shaanxi Province, China.

Since 1990s, intensive comprehensive preventive measures, mainly including rodent surveillance and control and free vaccination project, were performed, and HFRS cases have significantly decreased in most HFRS endemic areas of China [5]. Nevertheless, HFRS incidence has been rebounding drastically in recent years in some parts of China, such as Shaanxi Province, and becoming a severe public health problem again [8,9]. Baoji City is located in the Guanzhong Plain in the central part of Shaanxi Province (Fig 1), China, and the first HFRS case was reported in 1955. After that, Baoji has always been one of the most seriously affected areas in China despite the onset of vaccination in 1996 [10]. From 1996 to 2002, vaccination was carried out on the principle of voluntary payment. Free vaccination was performed among the people in the major HFRS areas of endemicity during 2003–2005 and among all the people since 2008. Totally, more than 1.26 million persons have been vaccinated with bivalent inactivated vaccine against both HTNV and SEOV since 1996. However, little is known about the epidemiology of HFRS in Baoji city. In addition, previous surveillance of orthohantaviruses infection humans and rodents were mainly performed to detect the virus or antibodies by indirect immunofluorescence [11]. Hence, the genetic characteristics of orthohantaviruses circulating in local are unavailable although such information is essential for laboratory diagnostics and virus surveillance in hosts.

To better understand the epidemiology of HFRS and associated pathogens from a public health perspective for humans, the epidemiological characters of HFRS were summarized and a molecular investigation of orthohantaviruses was performed in Baoji city in this study.

## Materials and methods

### Ethics statement

Collecting human serum samples from HFRS patients and rodent samples were carried out in accordance with the guidelines of the Baoji Center for Diseases Control and Prevention Institutional Committee and approved by the ethics committee of the Baoji Center for Diseases Control and Prevention. All rodents were treated in strict according to Guidance for Experimental Animal Welfare and Ethical Treatment by the Ministry of Science and Technology of China (http://www.most.gov.cn/fggw/zfwj/zfwj2006/200609/t20060930_54389.htm). The rodent animals were anesthetized with ether and prepared for surgery to collect the lung samples, and analgesics were used to minimize suffering under a protocol approved by the Baoji Center for Diseases Control and Prevention Institutional Animal Care and Use Committee (No. 2017011). Informed consents for blood sample collection and use in this study were obtained from all the HFRS patients under an approved human subject protocol by the Baoji Center for Diseases Control and Prevention Institutional Human Care and Use Committee (No. 2017012).

### HFRS cases data and sample collection

The records of HFRS cases occurring during 1984–2019 were obtained from the Baoji Center of Disease Control and Prevention.

In 2017 and 2018, sixty-three serum samples were collected from 63 HFRS patients diagnosed clinically in Baoji city, China. The antibodies (including both IgM and IgG) specific to orthohantaviruses were tested by Diagnostic kit for Antibody to orthohantavirus (Colloidal gold) (Xiamen Bosheng Biotechnology Co., Ltd., Xiamen, China).

To better understand the circulating pathogens of HFRS in rodents, 191 rodents were captured in March, June, September and December (one month from each season was selected) of both these two years in fields and residential areas using baited cages with a treadle release mechanism in Baoji city [12], Shaanxi Province, China, including Weibin, Jintai and Chencang districts, and Feng, Fengxiang, Qishan, Fufeng, Mei, Long, Qianyang, Linyou and Taibai counties, where most HFRS cases occurred in recent years based on the data from Baoji Center for Disease Control and Prevention. Lung tissue samples were collected under sterile conditions from all the trapped rodents and immediately stored in -80°C refrigerator for orthohantaviruses detection. All these rodents were initially classified into a specific rodent species by morphological examination. Subsequently, DNA was extracted from the lung sample of five randomly selected rodent samples from each species and all orthohantaviruses positive rodent samples to further confirm the rodent species by sequence analysis of the *mt-cyt b* gene [6].

### RNA extraction and detection of orthohantaviruses

According to the manufacturer’s instructions, total RNA was extracted from all collected rodent lung tissue samples using the TRIzol reagent (Invitrogen, Carlsbad, CA, USA) and HFRS patients’ serum using the TRIzol LS reagent (Invitrogen, Carlsbad, CA, USA), respectively. The RNA was dissolved with 30 μL RNase-free (DEPC-treated) water and stored in -80°C. The viral RNA was detected by amplifying partial (412-bp) large (L) RNA segment gene using nested reverse transcription PCR (nRT-PCR) described in previous study [6]. DEPC-treated ddH_2_O was used as negative control for PCR reactions and filter tips were also used to prevent contamination in each assay. Furthermore, each operation, including RNA extraction, PCR mixture preparation, template addition, and agarose gel electrophoresis, was performed in a fume hood in separate rooms, respectively.

### Amplification of partial/complete S gene

From all HTNV positive samples, 570-bp of the partial S gene sequence was also recovered using nRT-PCR with the primes as described previously with some modifications for primers [13]. In brief, primers (forward: GGRCAGACTGCWGAYTGG, reverse: ATCAATMARGCTTTGTGCC) were used in the primary round, and primers (forward: AACAAGAGGRAGGCARACAAC, reverse: AAGAAKGCYCCRAGTTCAGC) in the secondary. To obtain 564-bp of the partial S gene sequences of Dabieshan orthohantavirus (DBSV), primers DBSV-SW1 (GGGCARACTGCHGACTGG) and DBSV-SW2 (TCWGGRTCCATGTCRTCYCC) for the primary round and DBSV-SN1 (AACAAGRGGAAGRCARACRGC) and DBSV-SN2 (GCYCCCARYTCRGCAATRCC) for the secondary round were designed based on the conserved regions of known S gene sequences of DBSV. Moreover, complete S gene sequence was amplified using nRT-PCR with the prime HTV-MTF (TAGTAGTAGACTCCGCAARAAAIAS) described in previous study [14].

PCR reactions for amplifying partial S of DBSV were performed in 50 μL volumes. For the first round, the PCR mixture contained 25 μL Buffer (Takara, Dalian, China), 3 μL extracted total RNA, 2 μL for each primer (10 pmol), 2 μL Enzyme Mix and 16 μL water. Thermal cycling was performed with an initial cDNA synthesis at 50°C for 30 min, then denaturation at 94°C for 5 min, 30 cycles of denaturation at 94°C for 40 s, annealing at 56°C for 40 s, and elongation at 72°C for 1 min, and a final extension at 72°C for 7 min. For the second round, the PCR mixture contained 25 μL *Premix Taq* (Takara, Dalian, China), 3 μL first-round PCR products as template, 2 μL for each primer (10 pmol) and 18 μL water. The same thermal cycling condition without RT for cDNA synthesis was used as that in the first round.

### Sequencing of PCR products

PCR products of expected size were purified using TaKaRa MiniBEST Agarose Gel DNA Extraction Kit Ver.4.0 (TaKaRa, Dalian, China). After purified, the amplicons of partial L and S genes were subjected to direct sequencing in both direction using the ABI-PRISM Dye Termination Sequencing kit and the ABI 3730 genetic analyzer with the primers used in PCR. The purified DNA of complete S gene was ligated into pMD19-T simple vector and transformed into *E*.*coli* JM109 competent cells following the manufacturer’s instructions (TaKaRa, Dalian, China). The presence of insert was confirmed by PCR and subsequently sequenced using the ABI-PRISM Dye Termination Sequencing kit and the ABI 3730 genetic analyzer with universal M13-47 forward and RV-M reverse primers.

### Sequences analysis

The nucleotide sequences generated from each PCR product in this study were assembled and edited using Bioedit software [15], and the nucleotide identities were calculated using the ClustalW method implemented in the Lasergene program, version 5 (DNASTAR, Inc., Madison, WI). The best-fit nucleotide substitution model for phylogenetic analysis was determined by MEGA 6.0 [16]. The phylogenetic trees based on the partial L and S genes of orthohantaviruses were reconstructed using Bayesian method in MrBayes v3.1.2 [17]. In the Bayesian analysis, three hot and one cold Metropolis-coupled Markov Chain Monte Carlo (MCMC) chains were used and sampled every 100 generations to generate the trees. The MCMC algorithm was run until the average standard deviation of split frequencies less than 0.01 and Effective Sample Size (ESS) of all parameters more than 200 determined using Tracer Program [18]. Finally, the first twenty-five percent of sampled trees were discarded as burn-in. Meanwhile, the maximum-likelihood (ML) tree of partial S gene of orthohantaviruses was also evaluated using software PhyML v3.2 [19]. The tree was evaluated with 1000 replicates for bootstrap analysis. All the nucleotide sequences were submitted to GenBank under the GenBank accession numbers MK636780–MK636801 and MT542490–MT542511.

## Results

### Prevalence of HFRS in Baoji

**Fig 2:**
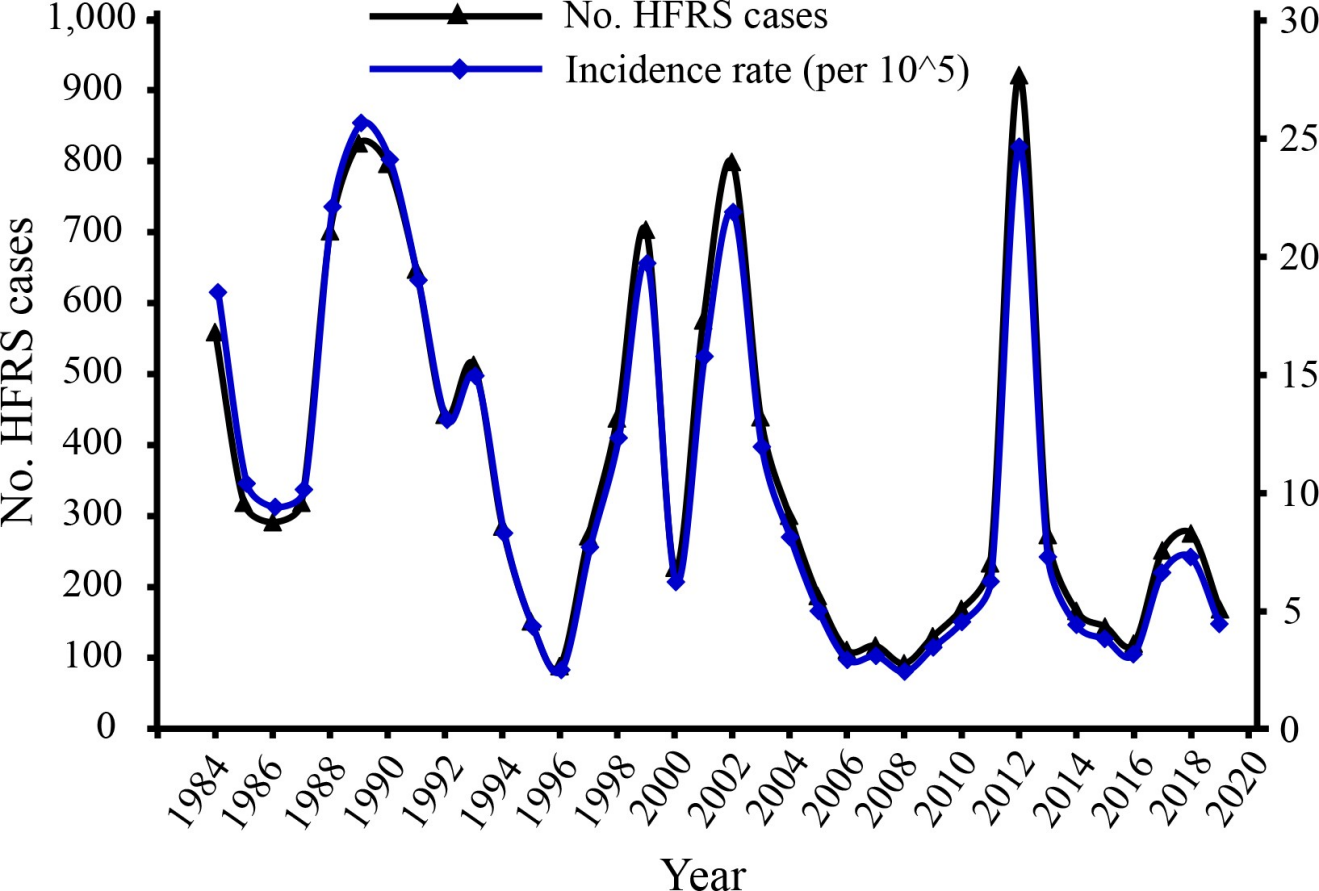
The annual number and incidence (cases/100,000 population) of HFRS during 1984–2019 in Baoji city, Shaanxi Province, China.

In Baoji, HFRS cases have occurred each year with a total of 13,042 cases and there were three major epidemics during the 36-year period between 1984 and 2019 (Fig 2). During 1988–1993, the first epidemic, with a total of 3,924 HFRS cases, occurred, and the number of cases reached a peak of 825 in 1989. Consistently, the incidence rate ranged from 13.01 to 25.65 cases per 100,000, and reached the peak in 1989. Five years later, the second epidemics appeared, and 3,180 cases occurred during 1998–2003 and a remarkably high peak of 799 cases was reached in 2002 (21.88 cases per 100,000). After these two epidemics, the annual number of HFRS cases decreased. Surprisingly, the number of HFRS cases increased dramatically in 2012 and formed the third epidemic with a total of 921 cases (24.65 cases per 100,000). Since 2016 to 2018, the number and annual incidence of HFRS cases increased gradually, and then decreased in 2019.

**Fig 3:**
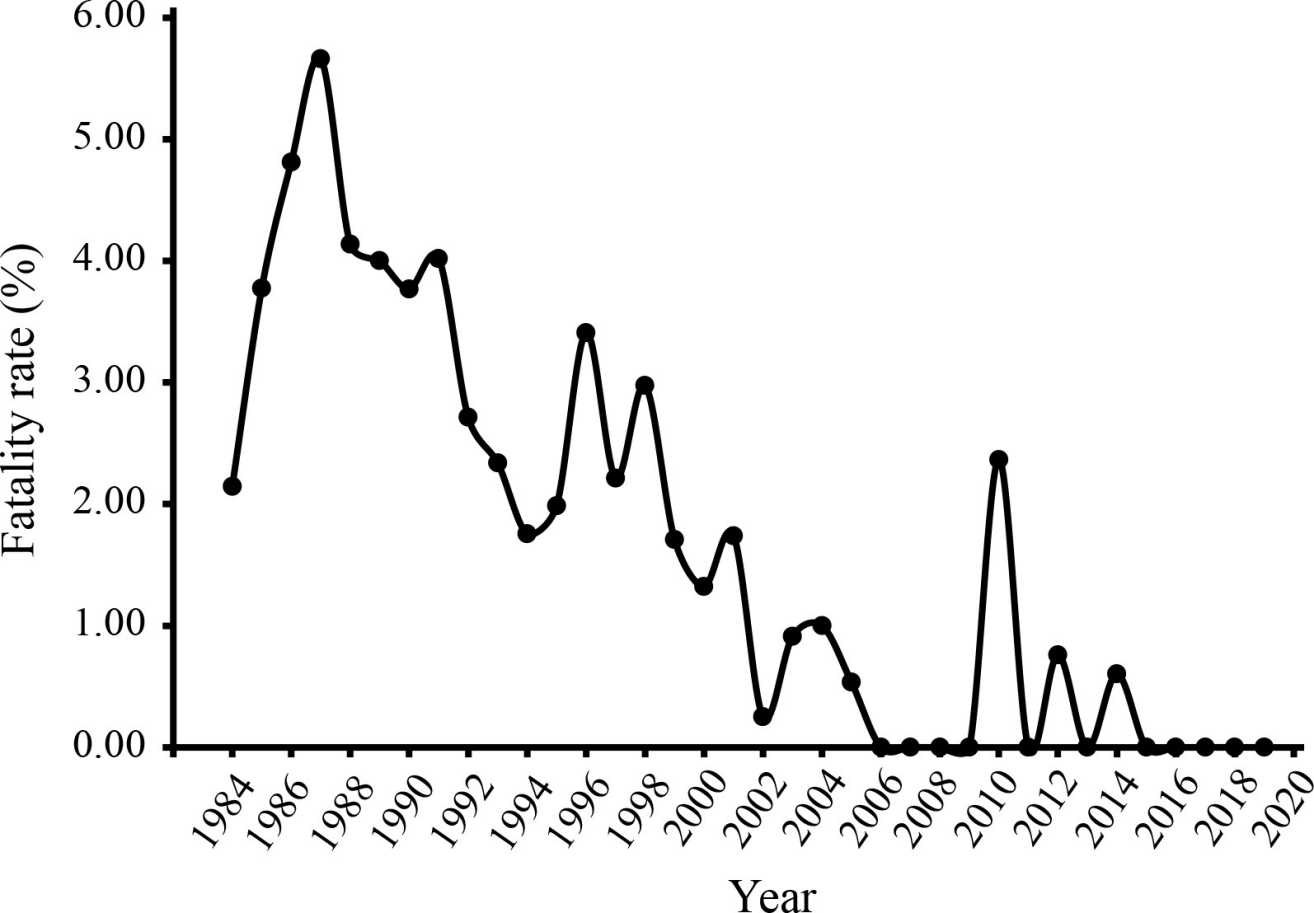
The mortality rate of HFRS during 1984–2019 in Baoji city, Shaanxi Province, China.

During 1984–2019, a total of 275 patients died due to HFRS in Baoji, with an average fatality rate of 2.10%. The average fatality rates reached 3.43% during the first 15 year period (1984–1998), and up to 5.66% in 1987. Since1999, the fatality rates decreased, and no death occurred in most years. However, 4 (2.37%), 7 (0.76%) and 1 (0.60%) fatalities were recorded in 2010, 2012 and 2014, respectively (Fig 3).

**Fig 4:**
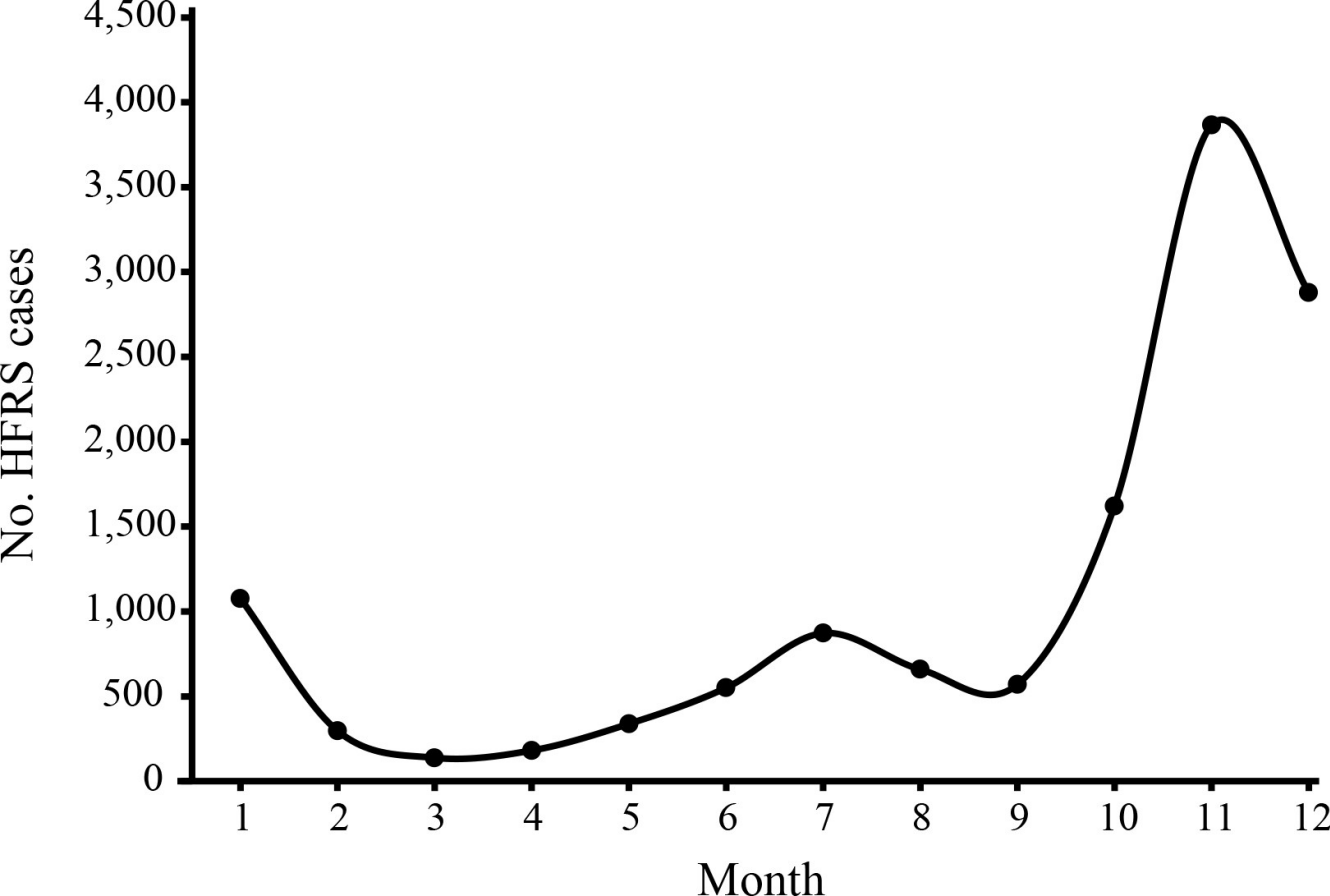
The monthly distribution of HFRS cases in Baoji during the period 1984–2019 in Baoji city, Shaanxi Province, China.

In addition, the seasonality of HFRS in Baoji was analyzed during the same periods. As shown in Fig 4, a sharp peak was observed in winter during November through January, corresponding with that mice-associated HFRS occurred mainly in winter [7]. However, a small peak also appeared from June to August.

### Orthohantaviruses identified in HFRS patients and rodents

Antibody tests based on colloidal gold immunochromatographic assay showed that eighteen serum samples from HFRS cases were positive for orthohantaviruses-specific IgM antibodies and others positive for both IgM and IgG antibodies.

By amplifying the partial L gene, PCR products of expected size were obtained from 12 of 18 above-mentioned only IgM positive human serum samples. Blast showed that all these sequences shared 96.2%–100% with each other and 85.2%–95.9% nucleotide identities with those from other known HTNV strains, suggesting that the virus detected from HFRS patients belonged to HTNV.

Species identification based on morphology showed that all sampled rodents belonged to six species, including *Apodemus agrarius*, *Rattus norvegicus*, *Niviventer confucianus*, *Rattus tanezumi*, *Mus musculus* and *Tscherskia triton* (Table 1). Furthermore, *mt-cytb* gene of five randomly selected rodent samples from each species and all orthohantaviruses positive rodent samples had the highest nucleotide identity (more than 99.0%) with those of above-mentioned six rodent species, respectively, consistent with the results of the morphological identification. *Apodemus agrarius* and *Mus musculus* were the dominant species in filed and residential areas, respectively. The number of rodents captured per season was 37, 52, 55 and 47, respectively.

**Table 1:** Prevalence of orthohantaviruses in rodents by species in Baoji, China*.

Species	Ecological location		Total
	Field	Residential area	
*Apodemus agrarius*	2/49	-	2/49 (2 HTNV)
*Rattus norvegicus*	1/7	1/26	2/33 (2 HTNV)
*Niviventer confucianus*	4/21	-	4/21 (4 DBSV)
*Rattus tanezumi*	0/10	-	0/10
*Mus musculus*	0/8	1/59	1/67 (1HTNV)
*Tscherskia triton*	1/7	0/4	1/11 (1 HTNV)
Total	8/102	2/89	10/191

*Data show no. PCR positive/no. of rodents captured

**Fig 5:**
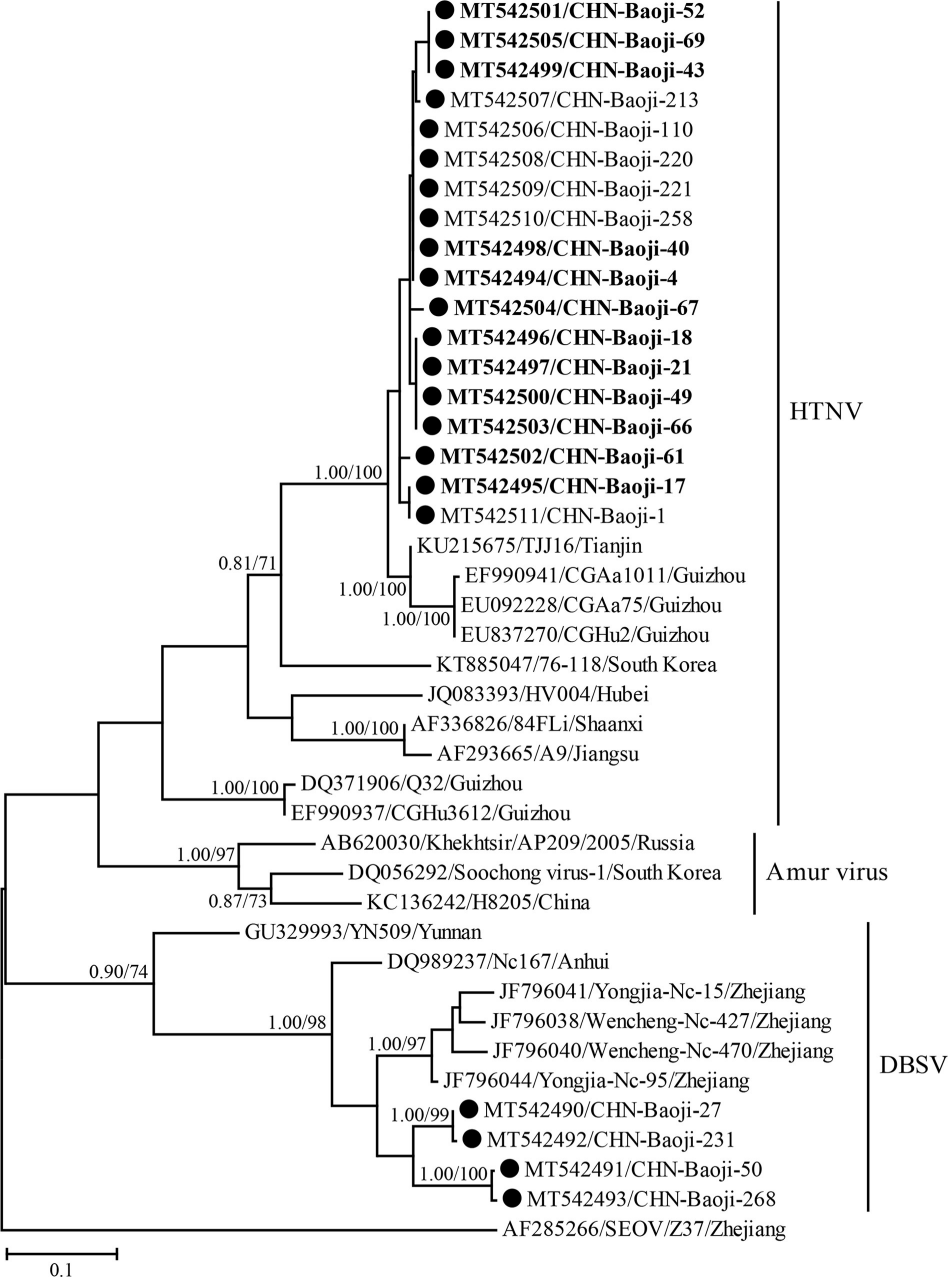
Phylogenetic tree based on the partial L gene segment sequences of orthohantaviruses using the Bayesian and ML methods. The strains obtained in the present study were marked by circles, with those collected from humans shown in Bold. Numbers at nodes indicated posterior node probabilities or bootstrap values and only >0.7/70 was shown. Seoul orthohantavirus (SEOV) was used as an outgroup. The scale bar represents the number of nucleotide substitutions per site.

PCR products with expected size by amplifying the partial L gene for orthohantaviruses detection were obtained from 10 lung tissue samples from 5 rodent species (Table 1), with an overall prevalence of 5.2%. Furthermore, 2, 1, 2 and 5 orthohantavirus positive rodent samples were distributed in each season, respectively. Blast showed that six sequences recovered from four rodent species (2 from *A*. *agrarius*, 2 from *R*. *norvegicus*, 2 from *T*. *tritonde* and *M*. *musculus*, respectively) shared 97.2%–100% nucleotide identities with each other and 84.9%–95.9% nucleotide identities with those from other known HTNV strains. In addition, all these novel variants fell within the diversity of HTNV on the partial L gene tree (Fig 5). Therefore, the virus was identified as HTNV. Interestingly, the partial L sequences of from HFRS patients and rodents were close related to each other, sharing 96.2–100% nucleotide identities. Additionally, other 4 strains from *N*. *confucianus* belonged to DBSV because their partial L gene presented 88.0%–92.6% nucleotide identities with those of other known DBSV strains and they clustered with other known DBSV strains on the partial L tree (Fig 5).

Besides partial L gene, partial S gene was also amplified successfully from all positive rodent samples. In addition, twelve patient serum samples tested positive for HTNV by detecting RNA using the same protocol. Meanwhile, four rodents were positive for DBSV based on the S gene, while no patient serum samples were found to be positive using the same protocol. All the newly generated partial S gene sequences of HTNV from both rodents and human samples were closely related to each other with less than 1.6% nucleotide divergence, and all these sequences in this study shared 84.2%–95.9% with those of other known HTNV strains in GenBank database. The four novel partial S gene sequences of DBSV from rodents in this study presented 90.2%–100% identities with each other and 81.4%–89.9% with those of other known DBSV strains in GenBank database. To better understand the genetic characteristics, complete S gene sequence was amplified successfully from CHN-Baoji-221, CHN-Baoji-50, CHN-Baoji-231, and CHN-Baoji-268 using nRT-PCR (MK636798–MK636801). The complete S gene sequence of CHN-Baoji-221 showed 86.7%–96.2% with those of other known HTNV strains, corresponding to 97.4 to 100% identity at the deduced amino acid level. Especially, these partial/complete S gene sequences of HTNV shared the highest nucleotide identities (with 3.8%–5.9% divergence) with strains TJJ16 in Tianjin city, YU61 in Henan province, and strains in Xi’an city and Guizhou province [14,20,21]. The complete S gene of CHN-Baoji-50, CHN-Baoji-231, and CHN-Baoji-268 of DBSV shared 91.9%–99.0% identities with each other, and 86.3%–91.9% with those of other known DBSV strains, corresponding to 98.1 to 100% identity at the deduced amino acid level.

### Phylogenetic analysis of partial S gene of Orthohantaviruses

**Fig 6:**
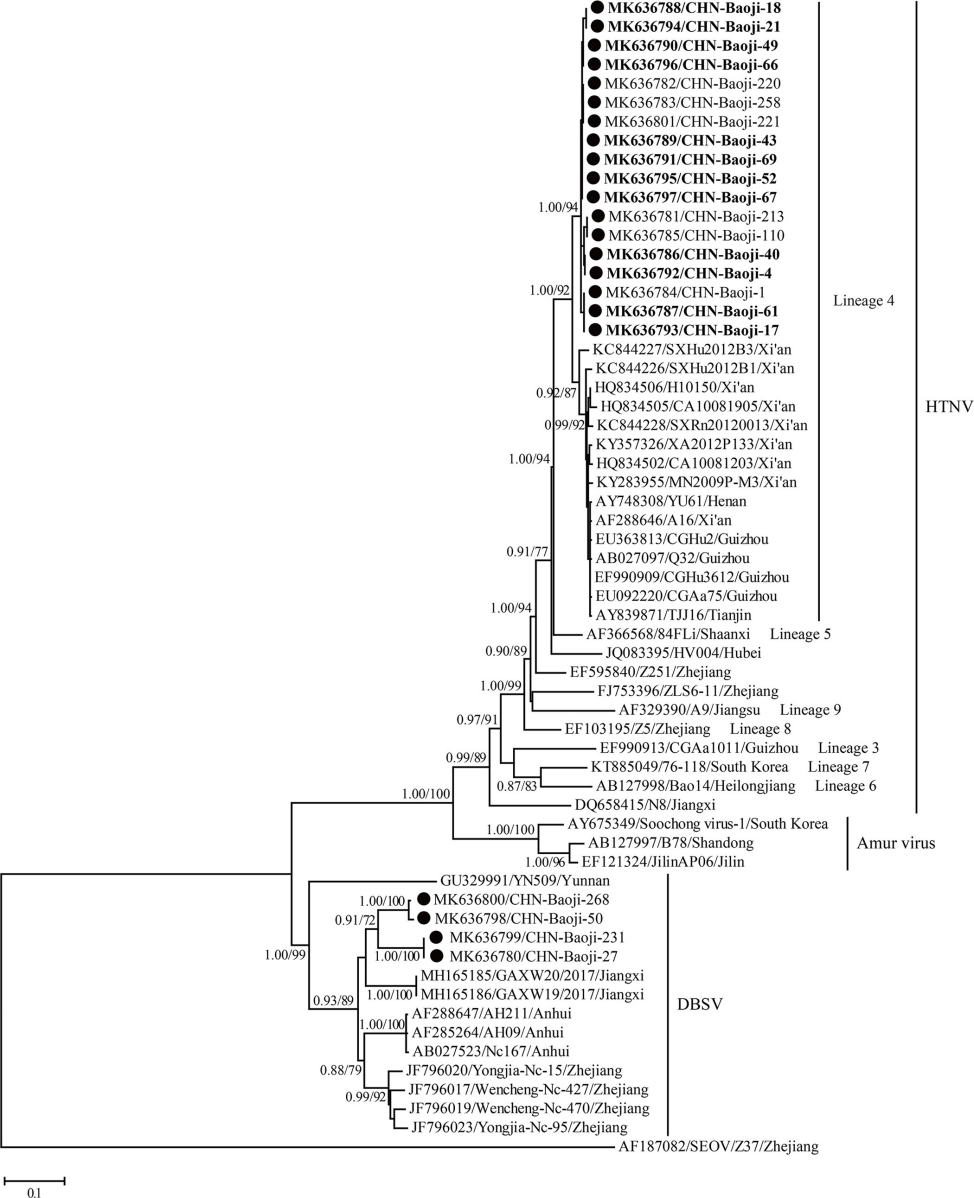
Phylogenetic relationships of orthohantaviruses identified in Baoji with known strains based on the partial S gene segment sequences using the Bayesian and ML methods. The strains obtained in the present study were marked by circles, and variants identified in humans are shown in Bold. Numbers at nodes indicated posterior node probabilities or bootstrap values and only >0.7/70 was shown. Seoul orthohantavirus (SEOV) was used as an outgroup. The scale bar represents the number of nucleotide substitutions per site.

GTR+Γ+G determined using MEGA 6.0 was the optimal nucleotide substitution model for S gene sequences data for phylogenetic analysis. Similar topology of partial S gene was observed based on the Bayesian and ML methods. In the partial S gene tree, all sequences of HTNV strains recovered in the current study formed a distinct clade although they were grouped into the fourth lineage (Fig 6) [14]. In addition, they were distantly related to other HTNV strains outside the fourth lineage, including 84FLi also isolated from human in Xi’an city, China. Within this lineage of HTNV, strains herein showed a closer evolutionary relationship with strains TJJ16, YU61, and strains identified in Xi’an city and Guizhou province, China, consistent with the similarity analysis [14,20,21]. Phylogenetic analysis based on the partial S gene showed that all DBSV, including the four novel DBSV strains, were divided into five well-supported clades. Interestingly, the strains herein formed two clades, and then clustered with those from Jiangxi province. They were distantly related to other known DBSV strains from Jiangxi, Zhejiang and Anhui provinces, especially YN509 from Yunnan province, China [14,22,23].

## Discussion

Consistent with the low incidence of HFRS in the whole country [5], the annual number of HFRS cases in Baoji has declined dramatically. However, high incidence of HFRS has been observed in several years, presenting higher HFRS cases in 1998, 2002 and 2012, and death cases occurred in 2010, 2012 and 2014, although comprehensive preventive measures for control of HFRS (including vaccinations since 1996) has been performed and living conditions of populations has been improved. In China, HFRS cases caused by mice-borne HTNV mainly occurred in winter and those by rat-borne SEOV in the spring and summer [7]. Hence, the seasonal analysis may provide important clues to the orthohantaviruses causing HFRS. In Baoji, the peak of HFRS emerged from November through January; therefore the pathogenic agent causing HFRS may be HTNV, which was confirmed by the molecular evidence of orthohantaviruses infection in humans and rodents. Interestingly, a small peak was also observed in summer, meaning that rat-associated SEOV may be the causative agent. However, SEOV was not identified in both humans and rodents. Hence, this small peak may be induced by other factors, such as ecology, human activities, and so on.

So far, 8 established and 3 tentative rodent associated orthohantaviruses were reported in China [5,14,24]. Of them, HTNV and SEOV are the pathogens of HFRS. Previous study showed that only HTNV were circulating in rodents and humans in Xi’an [21,25], a city also located in the Guanzhong Plain, about 200 kilometers away from Baoji. In this study, a survey of HFRS agents carried by rodents and infection in humans was performed for a limited period of time in Baoji city, China. The results revealed the prevalence of HTNV and DBSV in rodents, and HTNV in HFRS patients. However, no evidence for the existence of SEOV was found although its main host, *R*. *norvegicus*, was collected and SEOV was widely distributed in China and a small peak of HFRS was observed in summer. Hence, our limited results also suggested that the HTNV was the sole source of HFRS in Baoji, China, highlighting the need for improved surveillance on the natural infectious status of orthohantaviruses in rodents, especially those species presenting close contacts with humans. As it should be, we should strengthen the surveillance of SEOV in rats to determine whether it was circulating in local.

Since its first isolation in 1978, HTNV has been identified in several rodent species besides *A*. *agrarius*, including *A*. *peninsulae*, *Microtus fortis*, *R*. *norvegicus*, *M*. *musculus*, *N*. *confucianus*, *Tscherskia triton*, and so on, and suggested spillover occurred from natural host of HTNV to other rodent species [5,14]. In the current study, HTNV was identified from four rodent species suggesting host switching from *A*. *agrarius* to other species. More importantly, some HTNV sequences were recovered from rodent species close contacts with human, such as *R*. *norvegicus* and *M*. *musculus*, which may cause HTNV transmission from rodents to humans more easily although further study was needed to confirm this hypothesis [26]. Interestingly, high similarity, less than 1.6% divergence, shared by HTNV strains from both rodents and humans is indicative of direct viral transmission from rodents to humans.

Previous study showed that HTNV presented high genetic diversity, including at least nine genetic lineages [14]. Importantly, these divergent lineages displayed geographical clustering. In recent years, more studies suggested that the first and second lineages represented two novel species, DBSV and Amur virus (AMUV), respectively [22,23,27]. In addition, more novel lineages were reported in China [28]. In this study, the HTNV sequences presented 5.9% nucleotide divergence with those from the fourth lineage, and they formed a distinct clade in the partial S gene tree. Hence, both nucleotide similarity and phylogenetic analysis suggested that HTNV strains circulating in Baoji city belonged to a new clade in the fourth lineage [14].

DBSV was first isolated from *N*. *confucianus* in Anhui Province, China [14]. Recently, its homologous sequences were recovered from the same rodent species in Yunnan, Zhejiang and Jiangxi Provinces [22,23]. Furthermore, all these sequences formed distinct lineages based on the geographical pattern [22]. In the current study, phylogenetic analyses of the S gene sequences showed that the DBSV variants circulating in rodents in Baoji formed two novel DBSV lineages, and they were distantly related to other known strains. To date, all the DBSV variants were classified into six lineages including two ones found here, suggesting considerable genetic diversity of DBSV. More interestingly, this is the first report of DBSV in Shaanxi, enlarging its geographical distribution. Although the pathogenity of DBSV was unknown, we should pay more attention to it because it shared high similarity with HTNV and extensive genetic diversity and widely geographical distribution.

In sum, our results indicated that the changing incidence of HFRS in Baoji, China was observed. In addition, two orthohantaviruses, HTNV and DBSV, were circulating in rodents, and only the former was responsible for HFRS in Baoji, China. Hence, we should strengthen the surveillance of orthohantaviruses in rodents to control and prevent HFRS in Baoji, China.
